# Comparison of polymerase chain reaction and next-generation sequencing with conventional urine culture for the diagnosis of urinary tract infections: A meta-analysis

**DOI:** 10.1515/med-2024-0921

**Published:** 2024-03-28

**Authors:** Meng Zhao, Shuang Qi, Yinuo Sun, Xue Zheng

**Affiliations:** Department of Urology, First Affiliated Hospital, Heilongjiang University of Chinese Medicine, Harbin, Heilongjiang Province, 150001, China; Department of Pediatrics, Heilongjiang Provincial Hospital of Traditional Chinese Medicine, Harbin, Heilongjiang Province, 150001, China; Department of Nephrology, Harbin Jingen Nephropathy Hospital, Harbin, Heilongjiang Province, 150001, China

**Keywords:** urinary tract infections, polymerase chain reaction, next generation, sequencing, conventional urine culture, molecular diagnostic methods

## Abstract

The limitations of conventional urine culture methods can be avoided by using culture-independent approaches like polymerase chain reaction (PCR) and next-generation sequencing (NGS). However, the efficacy of these approaches in this setting is still subject to contention. PRISMA-compliant searches were performed on MEDLINE/PubMed, EMBASE, Web of Sciences, and the Cochrane Database until March 2023. The included articles compared PCR or NGS to conventional urine culture for the detection of urinary tract infections (UTIs). RevMan performed meta-analysis, and the Cochrane Risk of Bias Assessment Tool assessed study quality. A total of 10 selected studies that involved 1,291 individuals were included in this meta-analysis. The study found that PCR has a 99% sensitivity and a 94% specificity for diagnosing UTIs. Furthermore, NGS was shown to have a sensitivity of 90% for identifying UTIs and a specificity of 86%. The odds ratio (OR) for PCR to detect Gram-positive bacteria is 0.50 (95% confidence interval [CI] 0.41–0.61), while the OR for NGS to detect Gram-negative bacteria is 0.23 [95% CI 0.09–0.59]. UTIs are typically caused by Gram-negative bacteria like *Escherichia coli* and Gram-positive bacteria like *Staphylococci* and *Streptococci.* PCR and NGS are reliable, culture-free molecular diagnostic methods that, despite being expensive, are essential for UTI diagnosis and prevention due to their high sensitivity and specificity.

## 1 Introduction

Urinary tract infection (UTI) is an infectious condition that is frequently encountered in the adult population. Usually, these infections appear in the bladder or urethra. However, in more severe instances, they might impact the kidney [1]. Women exhibit a greater vulnerability to UTIs in comparison to men. Around 50–60% of women are projected to experience at least one UTI during their lifetime [2].

Bacterial infections are responsible for the majority of UTIs, and the standard therapy usually involves the use of antibiotics [3,4]. The healthcare industry bears substantial expenses for the treatment and management of UTIs, totaling billions of dollars annually, across both outpatient and inpatient settings [5]. The application of molecular testing techniques, such as next-generation sequencing (NGS) and polymerase chain reaction (PCR), for the identification and diagnosis of UTIs, has experienced substantial progress in recent years. The increase in popularity can be ascribed to the discontentment associated with the traditional method of exclusively depending on urine culture [6,7]. The accuracy of traditional culture methods in identifying acute UTIs is approximately 60%. The traditional approach of urine culture predominantly promotes the proliferation of rapidly growing aerobic bacteria, such as *Escherichia coli*, *Enterococcus*, and *Staphylococcus* species. Nevertheless, it is unable to adequately foster the majority of human commensal bacteria, which are distinguished by their slow growth, anaerobic nature, fastidiousness, or limited development in traditional cultures [8]. Therefore, the exact role of these bacteria in the development of UTIs is yet unknown. Molecular tools, such as NGS and PCR, have uncovered that the bladder has a diverse array of bacterial inhabitants, even in asymptomatic individuals who are in good health. PCR and NGS are culture-independent techniques used to identify and analyze microorganisms in a sample, thereby bypassing the limitations of standard urine culture methods [9]. The PCR test utilizes an advanced approach to duplicate a specific portion of DNA obtained from the patient’s urine sample. This replication process facilitates the identification of the particular pathogen accountable for the UTI, the determination of the most appropriate drugs for UTI treatment, and the evaluation of the bacteria’s resistance to different antibiotics. Qualitative PCR is used to determine the presence or absence of a pathogen, whereas quantitative PCR is performed to measure the amount of pathogen present [10,11]. NGS offers a thorough and detailed evaluation of the urine microbiome. Unlike PCR, which can only detect a limited number of organisms, NGS analyzes the complete microbial DNA in a urine sample and compares it to a comprehensive species database [12]. The application of these approaches has greatly improved our understanding of the urine microbiome and implicated these complex bacterial communities in the genesis of UTI symptoms. Recent studies have emphasized the use of molecular diagnostic tools like PCR and NGS to identify UTIs that are resistant to conventional urine culture techniques; as a result, clinical applications of commercial culture-independent diagnostic services like NGS and PCR are now widely available [13,14,15]. However, molecular diagnostic techniques are advocated for their enhanced sensitivity in detecting urine infections, but the efficacy of these strategies in this specific setting remains unknown. So, the aim of this meta-analysis is to compare how well culture-independent molecular diagnostic technologies like PCR and NGS work for diagnosing UTIs versus traditional urine culture. For this, relevant papers [15,16,17,18,19,20,21,22,23,24,25] selected as per the specific inclusion and exclusion criteria were used in this systematic review and meta-analysis.

## 2 Methods

The present study complied with the PRISMA (Preferred Reporting Items for Systematic Reviews and Meta-analyses) recommendations [26].

### 2.1 Eligibility criteria

The current study conducted an extensive examination of relevant academic articles published between 2000 and 2023. The PICO structure was employed to formulate specific selection criteria. In this context, P represented individuals with UTIs; I stood for the application of PCR and NGS for the detection of UTIs. The letter C represents the use of conventional urine culture for UTI detection, while the letter O encompasses clinical outcomes, the total number of positive UTI cases, and the microorganisms responsible for the infections. The studies examined and compared the diagnosis results of UTIs in patients using traditional urine culture methods and advanced molecular diagnostic methods such as PCR or NGS techniques. The researchers prioritized the inclusion of (1) full-text papers and (2) articles published in English in this meta-analysis. The abstracts were only included in the meta-analysis if sufficient information was provided. The analysis excluded papers that had inadequate data, lacked relevance to UTIs, or were published before 2000.

### 2.2 Information sources

The researchers performed a comprehensive and methodical examination of pertinent literature by searching the databases of MEDLINE/PubMed, EMBASE, Web of Sciences, and the Cochrane database, adhering to the guidelines specified in the Preferred Reporting Items for Systematic Reviews and Meta-Analyses (PRISMA).

### 2.3 Search strategy

The search was conducted using the following terms: “Urinary tract infections” or “UTI”; “Polymerase chain reaction” or “PCR”; “Next generation sequencing” or “NGS”; “meta-analysis”; “Causative agent of UTIs”; “Gram negative bacteria”; “Gram positive bacteria”; “Fungi”; “Protozoa”; “Conventional urine culture”; and “Molecular diagnostic methods.” The researcher conducted a comprehensive review of scholarly literature by utilizing the databases of PubMed and Cochrane libraries. In the context of searching Scopus, the title (ti)–abstract (abs)–keyword (key) field was utilized with the aforementioned keywords (Table A1). The key phrases “UTIs,” “conventional urine culture,” and “PCR and NGS for detection of UTI” were utilized in the Cochrane database. The integration of the Medical Subject Headings and textual keywords was accomplished by employing the Boolean operator “AND” within the context of the search strategy.

### 2.4 Selection process

The authors, MZ and SQ, conducted a comprehensive literature review to identify relevant studies. The researchers utilized inclusion criteria to exclude references that were outdated and to incorporate studies of significant relevance. In addition, two researchers conducted a thorough bibliographic search to identify pertinent and influential scholarly articles. A rigorous methodology was utilized to identify and incorporate pertinent studies published between the years 2010 and 2023

### 2.5 Data collection process and data items

The researchers MZ and SQ independently collected the demographic summary and event data from the studies included in this research. The main results were as follows: The study includes the following information: (1) the overall count of confirmed instances of UTI identified through traditional urine culture; (2) the overall count of confirmed instances of UTI identified through the molecular diagnostic techniques of PCR or NGS; (3) the types of Gram-positive and Gram-negative microorganisms implicated in UTI cases.

### 2.6 Sources of heterogeneity

The calculation of heterogeneity was performed among the experiments that were included. The Cochran *Q* statistic and the *I* ^2^ index were used in a random bivariate mode [27] and the RevMan software [28] was used to check for heterogeneity. Multiple sources of heterogeneity were investigated, encompassing the utilization of complete textual publications as opposed to abstracts, differences in age cohorts and sample sizes, variances in the bacteria evaluated, and differences in the outcomes of studies. Two reviewers, MZ and SQ, independently evaluated the methodological validity of the studies included in the analysis. The author XZ successfully settled any disputes that emerged between MZ and SQ through discussions and meticulous examination of data.

### 2.7 Risk of bias assessment

A pre-established, standardized questionnaire was used to assess the risk of bias in the articles that were considered for the analysis. The investigators utilized the Cochrane Risk of Bias: Robvis Tool [29] to produce a concise summary and visual representation illustrating the risk of bias.

### 2.8 Meta-analysis

The meta-analysis was performed utilizing the RevMan software (Review Manager, RevMan, Version 5, Copenhagen: The Nordic Cochrane Centre, The Cochrane Collaboration, 2020). The group exhibiting a degree of heterogeneity exceeding 50% opted to employ the random effect, while the subgroup with heterogeneity below 50% utilized the fixed effect. The primary methodology utilized in this study involved the application of the Mantel-Haenszel technique, which incorporated random bivariate effects. The aforementioned methodology was primarily utilized to calculate statistical measures such as sensitivity, specificity, and odds ratio (OR), along with a 95% confidence interval (CI) [30,31]. In addition, forest plots were generated to visually depict the aforementioned findings. The metrics used by the researchers to evaluate the extent of heterogeneity in the analyzed studies included tau^2^, chi^2^, *I* ^2^, and *z* values. Statistical significance was determined by considering a *p*-value below the predetermined threshold of 0.05. The DerSimonian and Lair method was utilized to compute the diagnostic OR using a 2 × 2 contingency table [32]. The evaluation of publication bias in the studies that were included in the analysis was performed utilizing Begg’s test [33] and Deek’s funnel plot [34]. The Deek’s funnel plot was constructed by plotting the logarithm of the OR for each individual study against its corresponding standard error, utilizing the MedCalc software [35].


**Statement of ethics:** An ethics statement is not applicable because this study is based exclusively on published literature.
**Study approval statement:** This study protocol was reviewed and approved by First Affiliated Hospital, Heilongjiang University of Chinese Medicine.

## 3 Results

### 3.1 Literature search results

**Figure 1:**
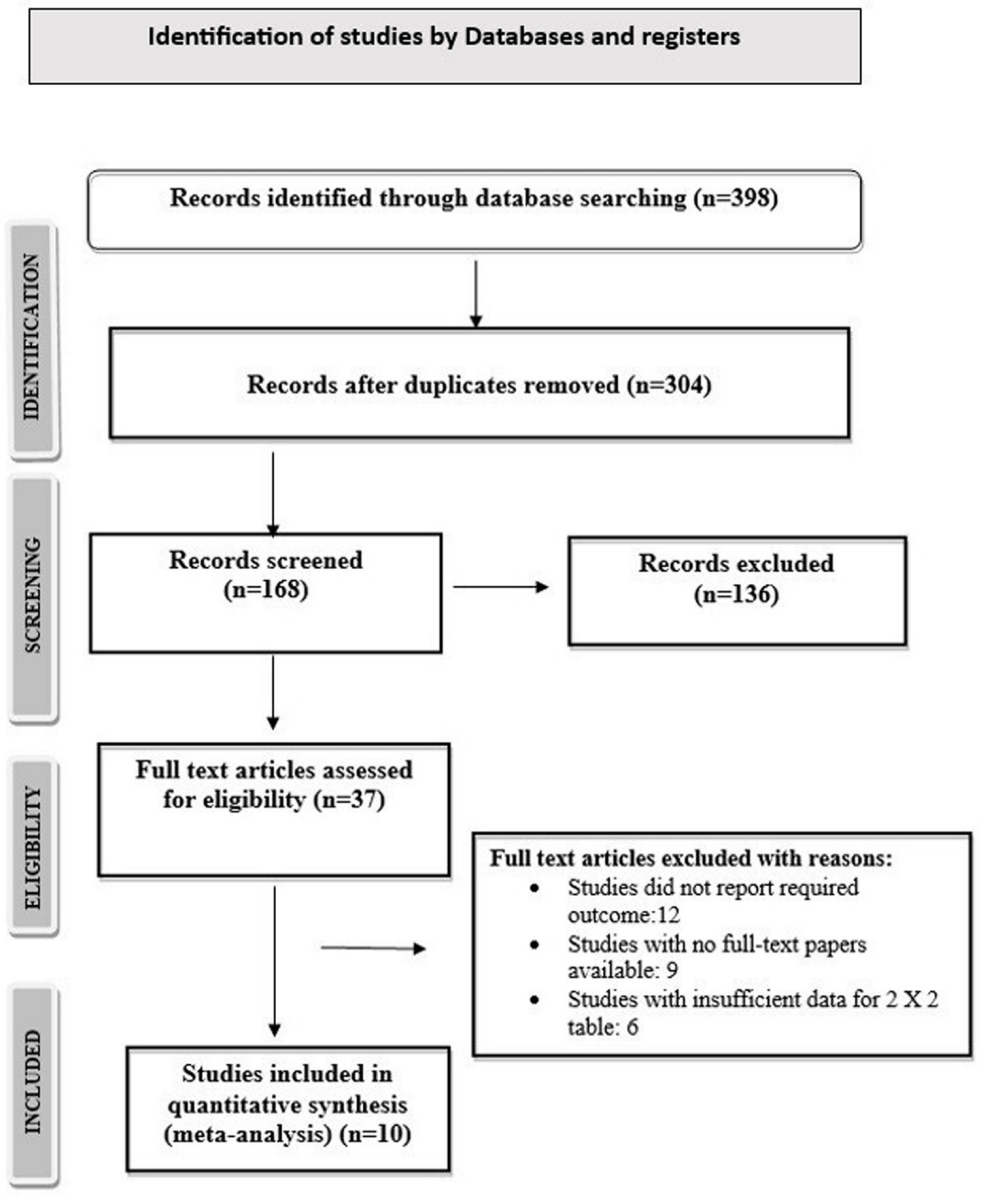
PRISMA flowchart of selection of studies.

The flowchart depicted in Figure 1 illustrates the utilization of the PRISMA framework in the process of selecting research studies. Following an extensive examination of online databases, a total of 398 studies were identified. After eliminating duplicate entries, a comprehensive set of 304 studies underwent a screening process based on the evaluation of their abstracts and titles. A thorough assessment was conducted on a total of 168 studies that met the predetermined criteria for inclusion. The present meta-analysis consisted of a total of ten studies, which were chosen according to pre-established criteria for inclusion and exclusion. The analysis included ten studies, of which five [16,17,18,19,20] evaluated the relative effectiveness of conventional urine culture and PCR in detecting UTIs, while the remaining studies examined the comparative efficacy of conventional urine culture and NGS for UTI detection. Table 1 provides a comprehensive summary of the relevant attributes of the studies that were included in the analysis pertaining to PCR. Conversely, Table 2 offers a comprehensive summary of the relevant attributes of the studies that were included in the analysis pertaining to NGS. The attributes encompass the identification of the studies, including their publication years, journals of publication, total number of UTI cases, age of patients, details of instruments and techniques employed, type of infection, molecular diagnostic method utilized, and identification of Gram-positive and Gram-negative bacteria responsible for the UTI.

**Table 1:** Characteristics of the included studies comparing PCR with conventional urine culture method

Study ID	Year of publication	Journal of publication	Total number of participants	Age of patients (years)	Type of infection	Molecular diagnostic method used	Instrument details	Conventional method used	Micro-organisms identified	
									Gram positive	Gram negative
Heytens et al. [16]	2017	Clinical Microbiology and Infection	220	≥18	UTI	PCR	DNA extraction: using Abbott Real-Time CT/NG assay (Abbott Laboratories) and Diagenode S-DiaMGTV (Diagenode Diagnostics, Seraing, Belgium) kit	Urine culture method	*S. saprophyticus*, *Trichomonas vaginalis*	*E. coli*, *Mycoplasma genitalium*
							PCR process: Abbott			
							m2000sp/rt instrument			
Ibraheam et al. [17]	2016	Pak. J. Biotechnol	30	≥18	UTI	PCR	DNA extraction: genomic DNA kit (Geneaid, China)	Urine culture method	*S. saprophyticus*, *Enterobacter*	*E. coli*, *P aeruginosa*, *Proteus mirabilis*
							PCR process: thermocycler Eppendorf programmed cycler			
Lehmann et al. [18]	2011	PLoS One	81	≥18	UTI	PCR	DNA extraction and PCR process: SeptiFastH, Roche Diagnostics	Urine culture method	*Staphylococci*, *Streptococcus*	*Escherichia coli*, *P. aeruginosa*, *Acinetobacter*, *Proteus mirabilis*
							GmbH, Penzberg, Germany)			
Wojno et al. [19]	2020	Infectious Diseases	582	≥18	UTI	PCR	DNA extraction: MagMAX DNA Multi-Sample Ultra Kit (ThermoFisher, Carlsbad, CA)	Urine culture method	*Streptococcus*, *Enterococcus*, *Actinobaculum schaali*	*Escherichia coli*, *Proteus* species, *Citrobacter* species, *P. aeruginosa*
							PCR process: Life Technologies			
							12K Flex OpenArray System.			
van der Zee et al. [20]	2016	PLoS One	211	≥18	UTI	PCR	DNA extraction: using E7526 kit, Sigma-Aldrich, Munich, Germany	Urine culture method	*Staphylococcus*, *Streptococcus*	*Escherichia coli*, *P. aeruginosa*, *Enterobacteriaceae*
							PCR process: ABI 7500 Real-Time PCR system (Applied Biosystems (ABI), Life Tech, Glasgow, UK)			

PCR: polymerase chain reaction; UTI: urinary tract infection.

**Table 2:** Characteristics of the Included studies comparing NGS with Conventional urine culture method

Study ID	Year of publication	Journal of Publication	Total number of cases	Type of Infection	Molecular diagnostic method used	Instrument Details	Conventional method used	Micro-organisms identified	
								Gram positive	Gram negative
Hasman et al. [21]	2014	Journal of Clinical Microbiology	35	UTI	NGS	Sequence analysis performed using MG-RAST.	Urine culture method	*E. faecalis*, *Lactobacillus*, or *Bifidobacterium*	*E. coli*, *Prevotella*, *Gardnerella*
Ishihara et al. [22]	2020	Drug Discoveries & Therapeutics	10	UTI	NGS	Sequence analysis performed using Genome Search Toolkit (GSTK)	Urine culture method	*Enterococcus faecalis*, *Aerococcus urinae*	*Escherichia coli*, *Proteus mirabilis*
McDonald et al. [23]	2017	Reviews in Urology	57	UTI	NGS	Sequence analysis performed using MicroGen DX (Orlando, FL)	Urine culture method	*Staphylococcus*, *Streptococcus*, *Enterococcus*, *Aerococcus urinae*, *Corynebacterium urealyticum*, *Enterobacter*	*Escherichia coli*, *Proteus* species, *Citrobacter* species, *P. aeruginosa*
Sabat et al. [24]	2017	Nature Scientific Reports	23	UTI	NGS	Sequence analysis performed using Nextera XT	Urine culture method	*Staphylococcus*, *Bacillus*	*Pseudomonas fluorescens*, *E. coli*, *Chryseobacterium*, *Enhydrobacter*, *Paracoccus*
						DNA Sample Preparation Kit (Illumina)			
Yoo et al. [25]	2021	Journal of Clinical Medicine	42	UTI	NGS	Sequence analysis performed using NEXTflex 16S V4	Urine culture method	*Staphylococcus*, *Streptococcus*, *Rothia*, *Enterobacteriaceae*	*Pseudomonas*, *Acinetobacter*, *Sphingomonas*
						Amplicon-Seq (BioO Scientific, Austin, TX, USA)			

UTI: urinary tract infection; NGS: next-generation sequencing.

### 3.2 Quality assessment of the included studies

**Figure 2:**
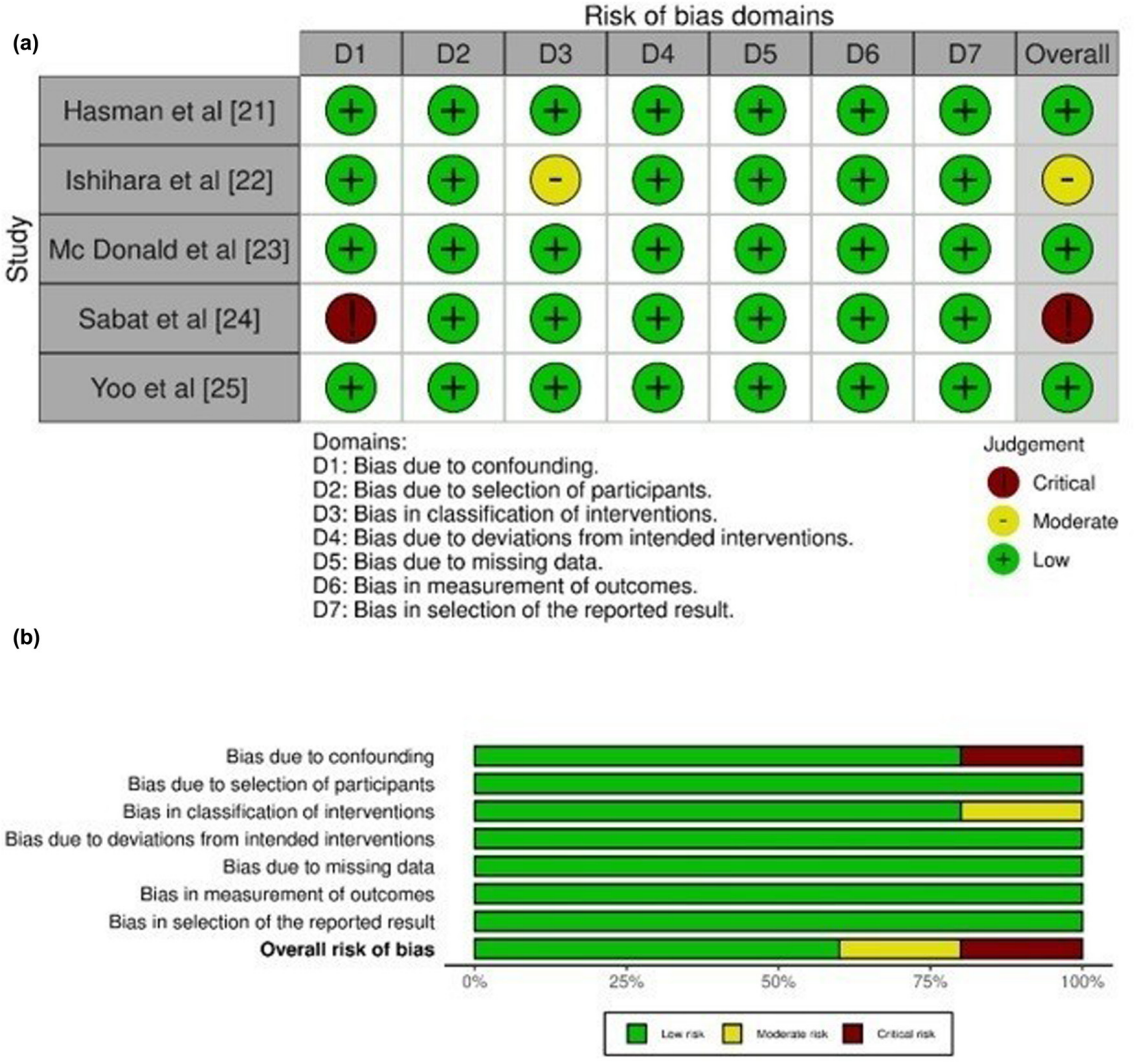
Risk of Bias analysis for studies related to NGS. (a) Risk of bias graph. (b) Risk of bias summary.

**Figure 3:**
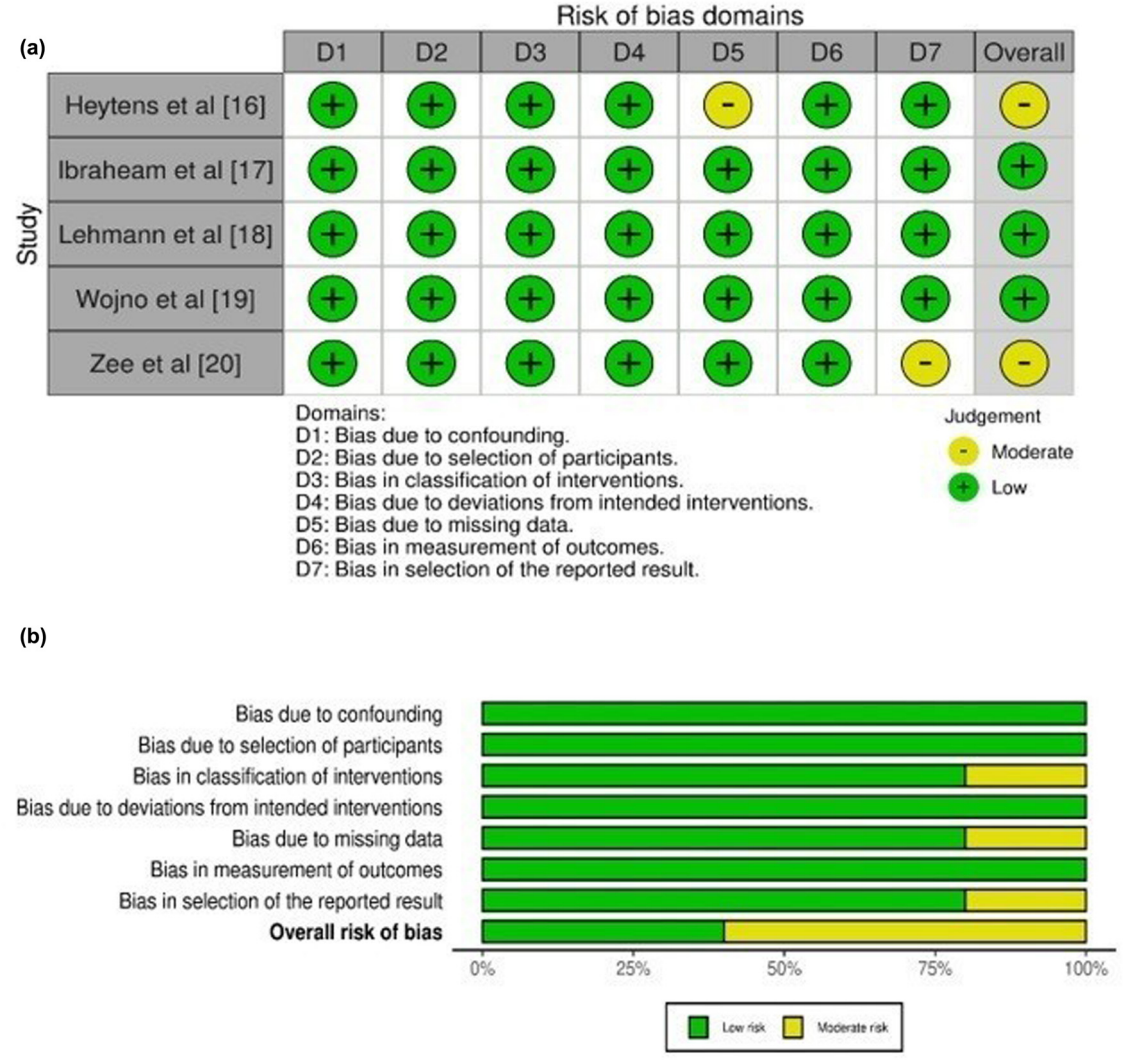
Risk of Bias analysis for studies related to PCR. (a) Risk of bias graph. (b) Risk of bias summary.

**Figure 4:**
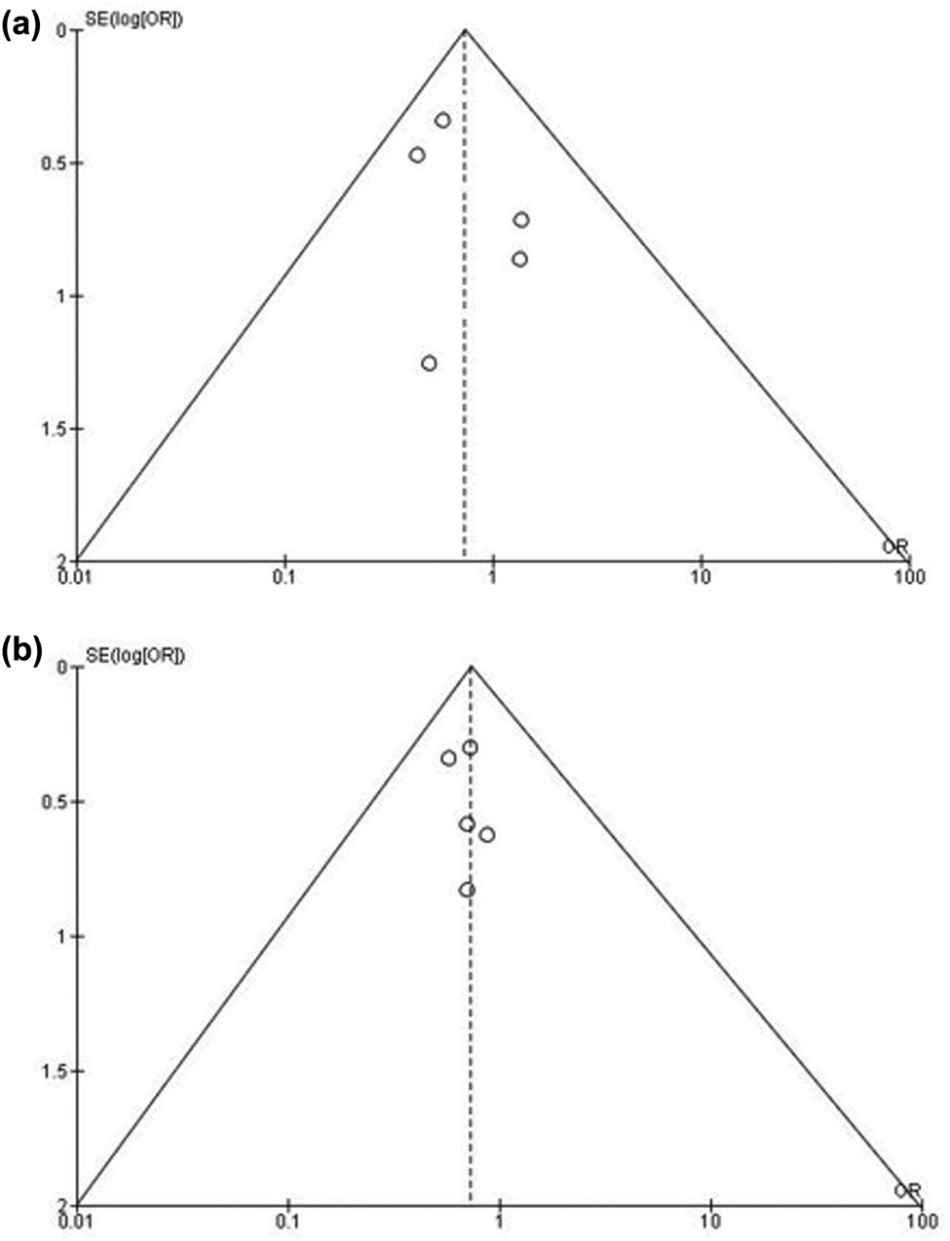
Funnel Plot for publication bias. (a) NGS; (b) PCR.

Table 3 displays the evaluation of the quality of the studies that were incorporated into this meta-analysis. Figure 2 provides a concise overview of the risk of bias assessment conducted for studies pertaining to NGS, while Figure 3 presents a succinct summary of the risk of bias analysis conducted for studies pertaining to PCR. The analysis for NGS incorporated five studies, of which one demonstrated a critical risk of bias resulting from confounding factors, while another exhibited a minimal risk of bias due to the classification of interventions. In a similar vein, the analysis for PCR included five studies, of which two were found to have a low risk of bias attributed to missing data and biased selection of reported results. The plot depicted in Figure 4 exhibits an inverted funnel shape for both PCR and NGS, suggesting the absence of publication bias [36]. This observation is further supported by the lack of statistical significance (*p* > 0.05) in the Begg’s tests for both NGS (*p* = 0.342) and PCR (*p* = 0.417) [37].

**Table 3:** Assessment of bias for included studies

	Heytens et al. [16]	Ibraheam et al. [17]	Lehmann et al. [18]	Wojno et al. [19]	van der Zee et al. [20]	Hasman et al. [21]	Ishihara et al. [22]	McDonald et al. [23]	Sabat et al. [24]	Yoo et al. [25]
Did the study avoid inappropriate exclusions	Y	Y	Y	Y	Y	Y	Y	Y	Y	Y
Did all patients receive the same reference standard	Y	Y	Y	Y	Y	Y	Y	Y	Y	Y
Were all patients included in the analysis	N	N	N	N	N	N	N	N	N	N
Was the sample frame appropriate to address the target population?	Y	Y	Y	Y	Y	Y	Y	Y	Y	Y
Were study participants sampled in an appropriate way?	Y	Y	Y	Y	Y	Y	Y	Y	Y	Y
Were the study subjects and the setting described in detail?	Y	Y	Y	Y	Y	Y	Y	Y	Y	Y
Were valid methods used for the identification of the condition?	Y	Y	Y	Y	Y	Y	Y	Y	Y	Y
Was the condition measured in a standard, reliable way for all participants?	Y	Y	Y	Y	Y	Y	Y	Y	Y	Y

Y –Yes; N – No.

### 3.3 Statistical analysis of the primary outcomes

The current meta-analysis comprised 10 research papers, which included a collective sample size of 1,291 individuals. The statistical analysis was conducted on the primary outcomes of the studies included in order to evaluate the effectiveness of culture-independent molecular diagnostic technologies, such as PCR and NGS, compared to conventional urine culture for diagnosing UTIs.

#### 3.3.1 Sensitivity and specificity of PCR for detection of UTI

**Figure 5:**
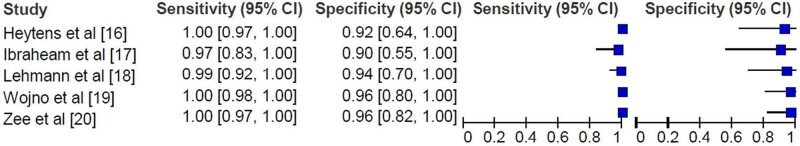
Forest plot for sensitivity and specificity of PCR for detection of UTI.

Figure 5 illustrates the sensitivity and specificity of PCR in detecting UTIs, as determined by analyzing data from five specific studies. These studies provided information on the number of positive UTI cases identified through both conventional urine culture and PCR, allowing for a comparative analysis. The combined sensitivity of PCR is 0.99 with a 95% CI of 0.82–1.0. Additionally, the combined specificity of PCR is 0.94 with a 95% CI of 0.55–1.0. The findings of this study indicate that PCR has a higher likelihood of detecting pathogens with greater specificity and accuracy when compared to the conventional culture method.

#### 3.3.2 Sensitivity and specificity of NGS for detection of UTI

**Figure 6:**
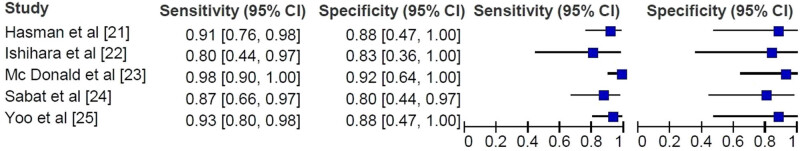
Forest plot for sensitivity and specificity of NGS for detection of UTI.

Figure 6 depicts the Sensitivity and specificity of NGS in the detection of UTIs, as determined through the analysis of data from five selected studies. These studies yielded data regarding the prevalence of positive UTI cases detected using both traditional urine culture methods and NGS, enabling a comparative examination. The aggregate sensitivity of NGS is 0.90, accompanied by a 95% CI ranging from 0.45 to 1.0. Moreover, the collective specificity of NGS is 0.86, accompanied by a 95% CI ranging from 0.35 to 1.0. The results of this study suggest that NGS exhibits a higher probability of identifying pathogens with increased specificity and accuracy in comparison to the traditional culture technique.

#### 3.3.3 UTI detection rate of PCR

**Figure 7:**
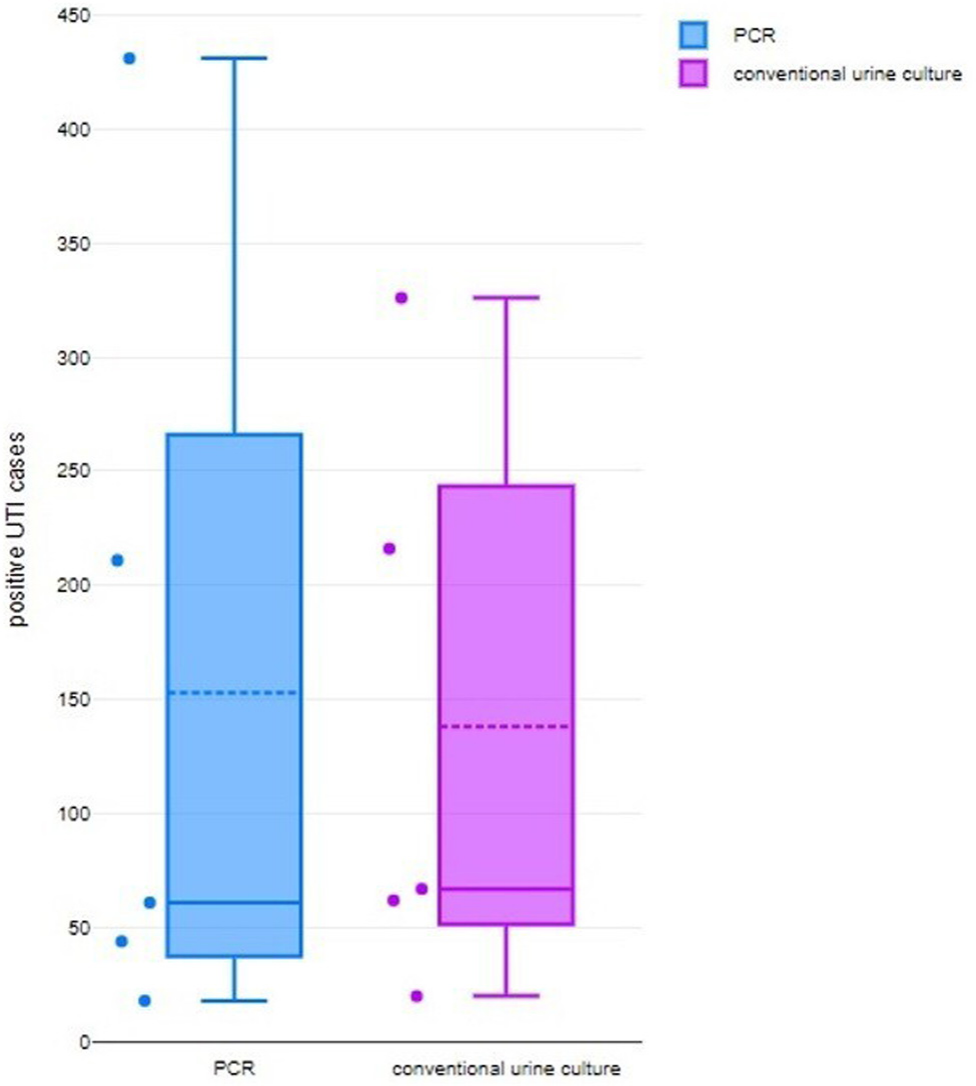
Box and Whisker plot comparing UTI detection rate by conventional urine culture vs PCR.

**Figure 8:**
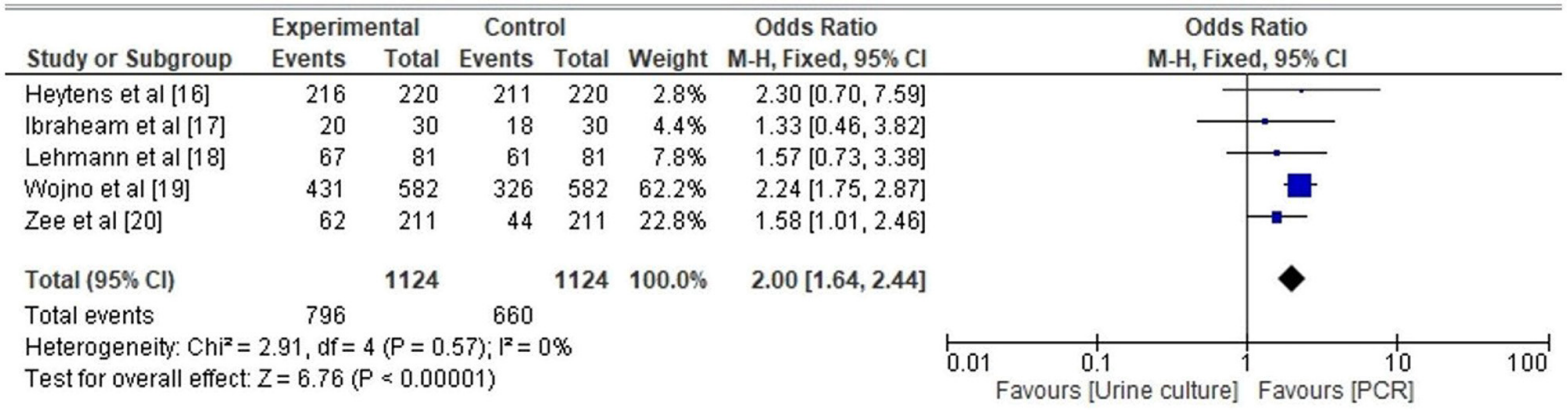
Forest plot for OR of detection of UTI by PCR.

Figure 7 depicts a Box and Whisker plot that demonstrates a discernibly higher rate of UTI detection using PCR method in comparison to the conventional urine culture method. The statistical parameters associated with the PCR technique encompass a minimum value of 20, a first quartile (Q1) value of 51.5, a median value of 67, a third quartile (Q3) value of 243.5, and a maximum value of 326. Additionally, the mean value is calculated to be 138.2, while the skewness coefficient is estimated to be 0.090, suggesting a distribution that may exhibit symmetry (*p*-value = 0.32). Furthermore, the distribution is characterized by a mesokurtic tail. In contrast, the traditional method of urine culture exhibits a minimum value of 18, a first quartile (Q1) of 37.5, a median of 61, a third quartile (Q3) of 266, a maximum value of 431, a mean of 153, a skewness of 1.38 suggesting a possibly symmetrical distribution (*p*-value = 0.128), and a mesokurtic tail. In an analogous way, the forest plot depicted in Figure 8 demonstrates that the likelihood of detecting UTIs through PCR is higher, as indicated by an adjusted odds ratio (AOR) of 0.50 [95% CI 0.41–0.61]. The findings exhibited heterogeneity, as indicated by the values of tau^2^ (0.00), chi^2^ (2.91), df (4), *I* ^2^ (75%), *z* (6.75), and *p* < 0.00001.

#### 3.3.4 UTI detection rate of NGS

**Figure 9:**
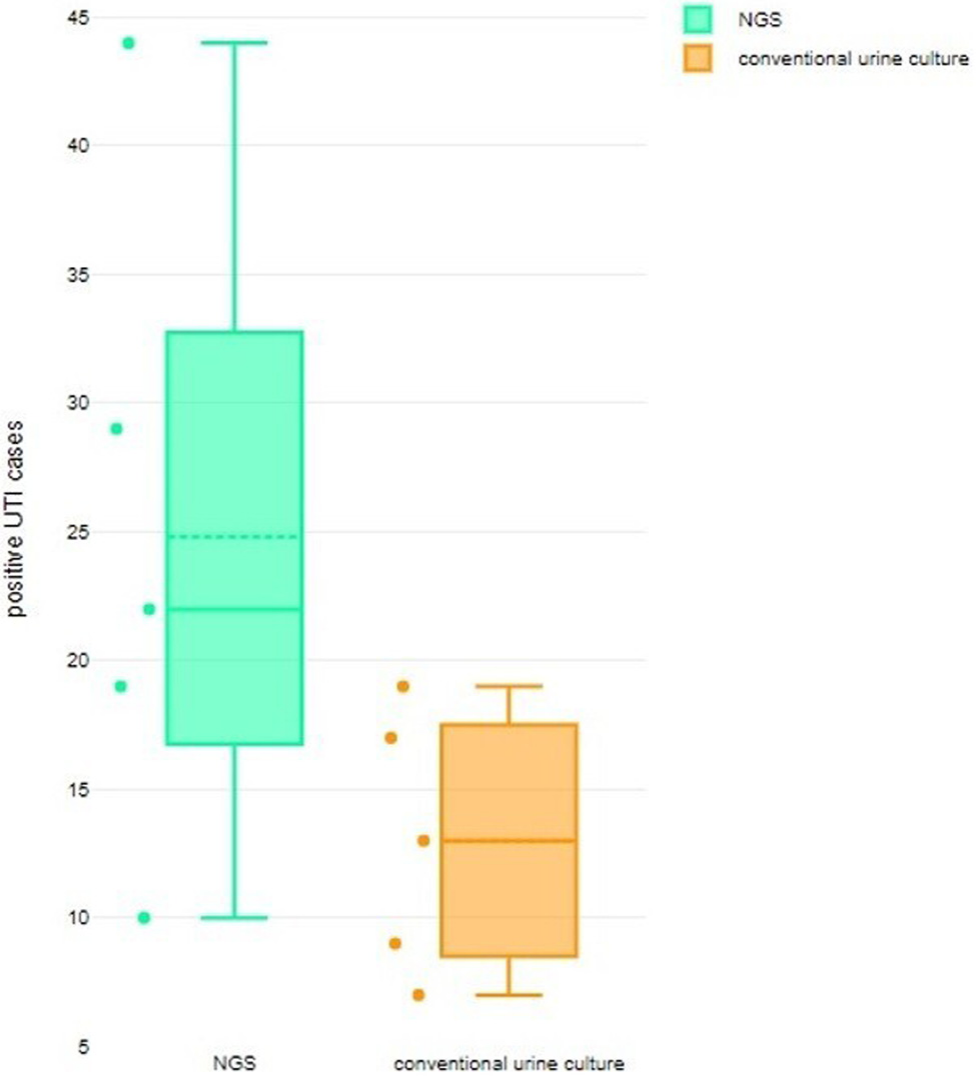
Box and Whisker plot comparing UTI detection rate by conventional urine culture vs NGS.

**Figure 10:**
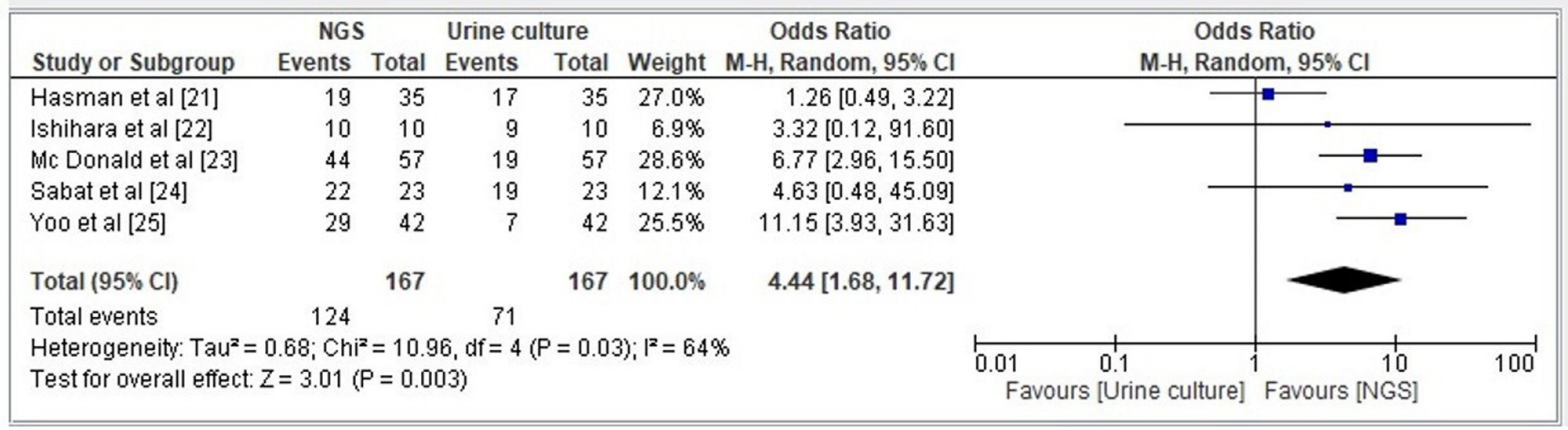
Forest plot for OR of detection of UTI by NGS.

The Box and Whisker plot presented in Figure 9 illustrates that the NGS method exhibits a notably higher rate of UTI detection compared to the conventional urine culture method. The statistical measures for the NGS method include a minimum value of 10, a first quartile (Q1) of 16.75, a median of 22, a third quartile (Q3) of 32.75, a maximum value of 44, a mean of 24.8, a skewness of 0.748371 indicating a potentially symmetrical distribution (*p*-value = 0.412), and a mesokurtic tail. On the other hand, the conventional urine culture method has a minimum value of 7, a Q1 of 8.5, a median of 13, a Q3 of 17.5, a maximum value of 19, a mean of 13, a skewness of 0.0 indicating a potentially symmetrical distribution (*p*-value = 1), and a mesokurtic tail. The forest plot displayed in Figure 10 illustrates that the likelihood of identifying UTIs using NGS is greater, as evidenced by an AOR of 0.23 [95% CI 0.09–0.59]. The results demonstrated heterogeneity, as evidenced by tau^2^ (0.68), chi^2^ (10.96), df (4), *I* ^2^ (64%), *z* (3.01), and *p* < 0.003.

#### 3.3.5 Pathogens detected in UTI by conventional urine culture, PCR, and NGS

The findings from conventional urine culture, PCR, and NGS analyses revealed that UTIs can be attributed to both Gram-positive and Gram-negative bacteria. The Gram-positive bacteria that have been identified include *Staphylococcus saprophyticus*, *Enterobacter*, *Staphylococci*, *Streptococcus*, *Enterococcus*, *Actinobaculum schaali*, *Lactobacillus*, or *Bifidobacterium*, *Enterococcus faecalis*, *Aerococcus urinae*, *Corynebacterium urealyticum*, *Bacillus*, and *Rothia.* Gram-positive bacteria, such as *Staphylococcus* and *Streptococcus*, have been found to be prevalent in the majority of UTIs. The Gram-negative bacteria included in this list are *Escherichia coli*, *Mycoplasma genitalium*, *Pseudomonas aeruginosa*, *Proteus mirabilis*, *Acinetobacter*, *Citrobacter*, *Prevotella*, *Gardnerella*, *Pseudomonas fluorescens*, *Chryseobacterium*, *Enhydrobacter*, *Paracoccus*, *Acinetobacter*, *Enterobacteriaceae*, and *Sphingomonas. E. coli* is the most commonly identified Gram-negative bacterium in the majority of UTIs. Other than this, the protozoa *Trichomonas vaginalis* was also responsible for causing UTIs (Table 4).

**Table 4:** UTIs causing microorganisms detected by PCR and NGS

Type of micro-organism	Name of micro-organisms detected
Gram-positive bacteria	*S. saprophyticus*, *Enterobacter*, *Staphylococci*, *Streptococcus*, *Enterococcus*, *Actinobaculum schaali*, *Lactobacillus*, or *Bifidobacterium*, *Enterococcus faecalis*, *Aerococcus urinae*, *Corynebacterium urealyticum*, *Bacillus*, and *Rothia*
Gram-negative bacteria	*Escherichia coli*, *Mycoplasma genitalium*, *Pseudomonas aeruginosa*, *Proteus mirabilis*, *Acinetobacter*, *Citrobacter*, *Prevotella*, *Gardnerella*, *Pseudomonas fluorescens*, *Chryseobacterium*, *Enhydrobacter*, *Paracoccus*, *Acinetobacter*, *Enterobacteriaceae*, and *Sphingomonas*
Protozoa	*Trichomonas vaginalis*

## 4 Discussion

In recent years, the utilization of molecular-based microbial profiling in the evaluation of UTIs has gained a substantial amount of relevance. PCR and NGS offer valuable diagnostic tools that have the potential to alleviate the ongoing cycle of aggravation and discomfort experienced by patients suffering from persistent UTIs [38]. NGS provides a highly complete assessment of the urine microbiome and examines the whole of microbial DNA present in a urine sample and subsequently compares it to a comprehensive database of species [39]. Similarly, the Urine PCR test distinguishes the existence of bacteria in a distinct manner. The utilization of a multiplex PCR) test enables the identification of a greater number of microbial species compared to the conventional urine culture method in individuals displaying symptoms indicative of a UTI [40]. The misapplication and misuse of antibiotics accelerates the development of antibiotic resistance [41]. PCR and NGS tests are characterized by their rapid detection rate, heightened sensitivity, and remarkable accuracy in identifying the bacteria responsible for UTIs. Consequently, these tests effectively tackle the problem of antibiotic resistance, enabling healthcare practitioners to provide informed recommendations regarding the appropriate choice of antibiotics and their optimal length of administration [42]. PCR possesses the capability to identify the presence of a pathogen responsible for symptoms, as opposed to genetic material of a microorganism that is clinically insignificant [43]. By amplifying specific segments of DNA, PCR enables the detection and characterization of target microorganisms at the species, strain, and serovar/pathovar levels. The method may also be employed to characterize whole populations of microorganisms in samples [44]. NGS offers in-depth strain genotyping and antibiotic resistance surveillance in some pathogens, such as Streptococcus pneumoniae, and is a very reliable predictor of antimicrobial-resistant status in others. Unlike PCR, NGS gives information about a sample’s entire set of genetic, regulatory, and biological properties. A urine culture can take up to seven days to provide results, whereas a PCR urine test has a quick turnaround time; results are often available in a day, and PCR costs roughly $5 per test. Simultaneously, the total turnaround time for identifying pathogens by mNGS testing is approximately 4 h, which is substantially faster than normal urine culture testing but slightly more expensive at $200 per test. Nevertheless, the utilization of PCR and NGS techniques remains valuable. However, it is important to acknowledge that traditional urine culture methods depend on the growth of live bacteria to identify species, whereas molecular-based techniques such as NGS and PCR do not [45]. The purpose of this meta-analysis was to investigate whether or not NGS and PCR are more accurate than the traditional approach of urine culture in identifying UTIs. Based on the findings of our study, it was found that both NGS and PCR techniques exhibit high levels of accuracy, sensitivity, and specificity in the diagnosis of UTIs. The study determined that the sensitivity of PCR in detecting UTIs was 99%, with a specificity of 94%. In contrast, the sensitivity of NGS in detecting UTIs was found to be 90%, with a specificity of 86%. The culture-free molecular-based methods have been found to exhibit a higher likelihood of detecting various types of Gram-positive and Gram-negative bacteria. This includes the accurate detection of Gram-positive bacteria such as *S. saprophyticus, Enterobacter, Staphylococci, Streptococcus, Aerococcus urinae, Corynebacterium urealyticum*, among others, as well as Gram-negative bacteria such as *Escherichia coli, Mycoplasma genitalium, Pseudomonas, Proteus mirabilis, Chryseobacterium*, and others. The prevalence of UTIs has been observed to be primarily associated with Gram-positive bacteria, including *Staphylococcus and Streptococcus*, as well as Gram-negative *Escherichia coli*. In line with our findings, Gasiorek et al. (2020) [46] conducted a review study in which they observed that molecular-based techniques, such as NGS and PCR, offer the potential to enhance patient assessment and management by effectively evaluating the urinary microbiome. In addition, Xu et al. (2021) [47], Dixon et al. (2020) [48], Szlachta-McGinn et al. (2022) [49], and Behzadi et al. (2019) [50] also advocate for the use of NGS and PCR techniques in the identification of UTIs). Nevertheless, there remains a need for novel methodologies to assess the merits and drawbacks of these contemporary and emerging diagnostic techniques, as well as to supplant the conventional urine culture method, which presently serves as the benchmark for diagnosing UTIs.

## 5 Limitations

The present investigation is limited by the diversity of PCR and NGS instruments as well as the variability of DNA extraction kit tools. Additionally, the involvement of different technicians introduces the potential for human error, thereby increasing the likelihood of false-negative outcomes. The present study exclusively focused on English-language publications, which potentially introduces a selection bias. Furthermore, the present analysis was executed with meticulous adherence to scientific protocols, and it is important to acknowledge that the findings are constrained due to the utilization of only 10 comparative studies characterized by varying degrees of heterogeneity, ranging from moderate to high levels. In the context of this meta-analysis, it would be advantageous to possess the capability to examine a diverse range of study-specific attributes that may be linked to the observed variations in reported outcomes and could provide further elucidation on the importance and effectiveness of culture-free molecular diagnostic-based PCR and NGS in the identification of UTIs.

## 6 Conclusion

Culture-independent molecular technologies such as NGS and PCR are widely utilized in the commercial diagnosis of UTIs due to reports of limited sensitivity of urine cultures. Increased sensitivity and specificity in the detection of urine bacteria are supported by moderate evidence. Therefore, in order to further substantiate these pieces of evidence, we conducted a comparative analysis between culture-independent molecular methods and conventional urine culture. The findings of a meta-analysis suggest that PCR and NGS exhibit considerable sensitivity in the detection of UTIs. However, further research is required to ascertain whether their routine application is supported by clinical implications and to compare patient symptoms and cure rates subsequent to antibiotic selection guided by molecular methods versus traditional urine culture.

## Abbreviations

UTIs: Urinary tract infections
PCR: Polymerase chain reaction
NGS: Next-generation sequencing
AOR: Adjusted odds ratio

## Appendix

**Table A1:** Database search strategy

Database	Search strategy
PubMed	#1 “Urinary tract infections” OR “UTI” OR “Polymerase chain reaction” [MeSH Terms] ^#^ OR “PCR” OR “Next generation sequencing” [All Fields] OR “NGS” OR “Gram positive bacteria” OR “Gram negative bacteria” [All Fields]” OR “Conventional urine culture” OR “Molecular diagnostic methods” [All Fields] OR “meta-analysis” OR “systematic review” [All Fields]
	#2 “Positive UTI cases,” OR “type of bacteria,” [MeSH Terms] OR “sensitivity,” OR “specificity,” OR “diagnostic odds ratio,” [All Fields]
	#3 #1 AND #2
Embase	#1 “Urinary tract infections” / exp^$^ OR “UTI”/ exp OR “Polymerase chain reaction”/exp OR “PCR”/exp OR “Next generation sequencing”/ exp OR “NGS”/ exp OR “Gram positive bacteria” /exp OR “Gram negative bacteria”/ exp OR “Conventional urine culture” / exp OR “Molecular diagnostic methods”/exp OR “meta-analysis”/exp OR “systematic review”/exp
	#2 “Positive UTI cases” / exp OR “type of bacteria”/ exp OR “sensitivity”/ exp OR “specificity” /exp OR “diagnostic odds ratio” exp
	#3 #1 AND #2
Cochrane library	#1 ( Urinary tract infections): ti, ab, kw^@^ OR ( UTI): ti, ab, kw OR ( Polymerase chain reaction): ti, ab, kw OR ( PCR): ti, ab, kw OR ( Next generation sequencing): ti, ab, kw OR ( NGS): ti, ab, kw OR ( Gram positive bacteria): ti, ab, kw OR ( Gram negative bacteria): ti, ab, kw OR ( Conventional urine culture):OR ( Molecular diagnostic methods): ti, ab, kw OR (meta-analysis) ti, ab, kw (Word variations have been searched)
	#2 (Positive UTI cases): ti, ab, kw OR (type of bacteria): ti, ab, kw OR (sensitivity): ti, ab, kw or (specificity): ti, ab, kw or (diagnostic odds ratio): ti, ab, kw (Word variations have been searched)
	#3 #1 AND #2

#MeSH terms: Medical Subject Headings; $ exp: explosion in Emtree- searching of selected subject terms and related subjects; @ ti, ab, kw: either title or abstract or keyword fields.
